# Use of Continuous Intravenous Anakinra Infusion in Multisystem Inflammatory Syndrome in Children

**DOI:** 10.1155/2023/8530060

**Published:** 2023-02-28

**Authors:** Abdulaziz Alolayan, Abdullah S. Aldamegh, Azzah Alkhayrat

**Affiliations:** ^1^Pharmacy Department, Prince Sultan Military Medical City, Saudi Arabia; ^2^Pediatrics Department, Unaizah College of Medicine and Medical Sciences, Al-Qassim University, Saudi Arabia

## Abstract

Coronavirus disease (COVID-19) is an emergency pandemic with a high mortality rate worldwide. One of its complications in children is developing multisystemic inflammatory syndrome related to cytokine storm. Anakinra is a recombinant human interleukin-1 (IL-1) receptor antagonist used to suppress the exaggerated inflammatory response in such conditions, and it is potentially lifesaving in a cytokine storm. We present the case of a patient with critical COVID-19 associated with multisystem inflammatory syndrome in children (MIS-C) successfully treated with anakinra intravenous (IV) infusion.

## 1. Introduction

During the COVID-19 pandemic, there has been an increase in reports of the pediatric severe acute respiratory syndrome (SARS) related to multi-inflammatory disorders. Moreover, it is known as MIS-C associated with COVID-19, which shares some clinical characteristics with Kawasaki disease (KD) [1–3]. Kawasaki disease's clinical characteristics are persistent fever, bulbar conjunctivitis, skin rash, or mucosal involvement [4]. They present with respiratory and gastrointestinal symptoms in the form of severe disease action. At the same time, some cases are ending in macrophage activation syndrome (MAS) [5–10]. It has implied that the syndrome results from hyperinflammation caused by a cytokine storm [11]. Anakinra is an IL-1 receptor antagonist used for some rheumatoid diseases and other inflammatory conditions [12, 13]. Updated published data announced the effectiveness and safety of anakinra in reducing mortality and acute care admission in patients with COVID-19 associated with MIS-C. Anakinra dose studied for MIS-C is from 5-10 mg/kg/day subcutaneously (SC) daily or in divided doses, and higher doses might be considered [14–17]. Some studies showed that the feasibility of using IV administration of anakinra instead of SC supported its efficacy and safety [18, 19]. Monteagudo et al. demonstrated that the utilization of continuous IV anakinra infusion dose reached up to 2400 mg/day resulting in rapid serologic and subsequent clinical improvement in adult patients with MAS. Their study notified that anakinra has short and unclear stability in solution based on the manufacture leaflet. Therefore, the prepared solution was infused over a maximum of 8 hours [20]. Due to the lack of evidence, we report our experience using continuous IV infusion of anakinra for refractory MIS-C in a tertiary care hospital center.

## 2. Case Report

On January 16, 2022, a fourteen-month girl was brought to the emergency room of Prince Sultan Military Medical City (PSMMC) with a history of fever and cough for four days that worsened in the last two days and was associated with a decrease in the level of activity. She had a positive history of contact with sick family members with respiratory symptoms; however, it was not investigated for COVID-19. The fever initially subsided by paracetamol IV (15 mg/kg/dose) every 6 hours and then became persistent, ranging from 39–40°C. She has a known case of craniosynostosis, gastroesophageal reflux disease, and global developmental delay that are under investigation. Infection with COVID-19 was confirmed by reverse transcription polymerase chain reaction (RT-PCR) on the nasopharyngeal swab, and chest X-ray (CXR) showed infiltration mainly on the left side. The lab results are shown in Table 1.

The patient initially had been inserted on noninvasive ventilation, “high flow nasal cannula,” supportive care and started on antimicrobials amoxicillin-clavulanic acid IV (90 mg/kg/day) as empirical therapy until full investigation become resulted and remdesivir IV (5 mg/kg as loading dose and then 2.5 mg/kg/daily) for viral infection. On day two, the patient had been successfully weaned from the nasal cannula (NC). However, later, the patient's condition had been worsened as oxygen saturation reached 78%, and CXR showed a collapse in her left lower lob and consolidation of the right mid and lower zones. Therefore, she had been admitted to the pediatric intensive care unit (PICU) and started on continuous positive airway pressure (CPAP) at 8 and fractional inspired oxygen (FiO2) at 100%; antibiotic was upgraded from amoxicillin-clavulanic acid to piperacillin-tazobactam IV (300 mg/kg/day) to treat hospital-acquired infection because the patient's condition was seemed to be a picture of sepsis. CXR was repeated and showed bilateral collapse. CPAP increased to nine, and FiO_2_ was 100%. Later, the patient's oxygen was desaturated (75%-80%), with significant suprasternal and subcostal retractions. As a result, she had been intubated through high-frequency oscillatory ventilation (HFOV) because the patient's condition deteriorated with a picture of acute respiratory distress syndrome (ARDS). The patient had been paralyzed and sedated. Antibiotics were upgraded to meropenem IV (60 mg/kg/day) and vancomycin IV (60 mg/kg/day), and the rheumatology team had been consulted for possible complications of COVID-19 with MIS-C. Methylprednisolone pulse therapy had been suggested to start and was already given (30 mg/kg) by IV due to the deterioration of the patient's condition.

On day three, systolic blood pressure dropped to 50, diastolic to 28, and mean arterial pressure became 37. Vasopressors had been given for one day only. An echocardiogram was done and showed moderate pericardial effusion, and pro-BNP reached 2093 pg/mL. MIS-C was confirmed based on the lab results and clinical condition. IV immunoglobulin (IVIG) had been given on day three for a total of two doses. Methylprednisolone pulse therapy had been completed for five days and then shifted to maintenance dose. On day four, the patient's condition still had not improved. So the rheumatology team recommended to start anakinra SC (8 mg/kg/day), and by the next day, the dose had been titrated up, and the route of administration shifted to IV push. On day 10, the patient still had not improved. Based on Monteagudo et al.'s study, the dose and the route of anakinra had been shifted from IV push to IV infusion to (12 mg/kg/day) over 24 hours. Furthermore, due to the instability of IV preparation, the medication had been prepared in separate bags to be run for 8 hours and diluted in sodium chloride 0.9%. On day 14, another corticosteroid pulse therapy had been given for three days. On day 22, the patient's condition had been improved, and the respiratory therapy had been shifted from HFOV to synchronized intermittent mandatory ventilation(SIMV). On day 25, the patient was extubated to NC. A sharp reduction of inflammatory markers and ferritin is observed in Table 1.

Respiratory parameters had been improved, followed by a favorable radiographic evolution. On day 26, the patient discharged from the ICU. Tapering of methylprednisolone dose was planned, and anakinra shifted to the SC (4 mg/kg/daily). Recently, the patient was discharged on room air and has been followed up with the rheumatology team as outpatient clinic. During the ICU admission, full panel of septic work up had been requested including virus PCRs, and patient infected only with *Stenotrophomonas maltophilia* through tube as secondary to COVID-19 infections, and treated with co-trimoxazole IV (20 mg/kg/day). Meropenem and vancomycin were discontinued after finding the final results. Antifungal also had been started from day seven for eight days to rule out fungal infection due to worsening condition.

## 3. Discussion

To our knowledge, this is the first report of a severe case of COVID-19 related to MIS-C effectively treated with anakinra IV infusion. Lately, a cytokine storm has been associated with the critical stage of COVID-19, resulting in hyperinflammatory effects, with suspicion of poor prognosis [21]. Hyperinflammatory syndrome is described by life-threatening hypercytokinemia leading to multiorgan failure. A retrospective multicenter study found that the mortality rate of 190 confirmed COVID-19 cases in Wuhan was associated with COVID-19-driven hyperinflammation [22]. In cytokine storms, IL-1*β* appears to be one of the significant proinflammatory markers. Anakinra is an antagonist of the IL-1*β* receptor, which inhibits the release of IL-1*β* from macrophages. Schmitz et al.'s study shows that the blockage of IL-1 has been associated with the reversal of acute lung injury in avian influenza [23]. Fingerhutová et al.'s study demonstrated that aggressive therapy of MAS using continuous IV anakinra infusions in higher doses successfully reverses the cytokine storm, especially in the pediatric population [24]. In the case of our patient, treatment with different modalities like upgrading antibiotics, two courses of corticosteroid, IVIG, and SQ with IV push of anakinra did not provide clinical improvement immediately. In contrast, using anakinra IV infusion with a higher dose showed a synergic effect in improvement. However, a possible benefit due to the late effect of these modalities, late effect cannot be ruled out. Although the solid evidence-based studies of anakinra did not test the feasibility of using IV infusion, our case report showed promising efficacy of anakinra IV infusion, and no severe side effects appeared during ICU admission.

## 4. Conclusion

During COVID-19, whether moderate or severe, there are many challenges in the way of therapeutic plans, and using anakinra is one of them. Our case suggests that administering IV infusion of anakinra in cytokine storm related to MIS-C contributed to a significant clinical improvement, is workable, and has a safe profile.

## Figures and Tables

**Table 1:** Laboratory characteristics of patient.

	Day 1	Day 3	Day 14	Day 26
	Hospital admission	Pediatric ICU admission	2^nd^ steroid pulse therapy	Discharge from pediatric ICU
WBC count (×109/L)	3.20	6.0	16.9	13.34
Lymphocyte count (×109/L)	1.16	1.43	7.1	2.77
Hemoglobin (g/L)	11.5	9.1	9.2	10.2
PLT count (×109/L)	60	95	178	224
Ferritin (mg/L)	2028	901	6933	413
CRP (mg/dL)	141	34	8.52	7.72
AST (U/L)	526	195	112	44
ALT (U/L)	206	80	114	36
GGT (U/L)	242	227	207	430
LDH (U/L)	>1800	1295	602	281
Fibrinogen (g/L)	5.3	2.5	4	3.9
D-dimer ng/mL)	4630	2080	2960	520

## Data Availability

There is no data availability to be reached. All of the information is supplied in the manuscript.
